# Pore formation by human stefin B in its native and oligomeric states and the consequent amyloid induced toxicity

**DOI:** 10.3389/fnmol.2012.00085

**Published:** 2012-08-02

**Authors:** Gregor Anderluh, Eva Žerovnik

**Affiliations:** ^1^National Institute of ChemistryLjubljana, Slovenia; ^2^Department of Biology, Biotechnical Faculty, University of LjubljanaLjubljana, Slovenia; ^3^IPS Research Group, Jožef Stefan International Postgraduate SchoolLjubljana, Slovenia; ^4^Department of Biochemistry and Molecular and Structural Biology, Jožef Stefan InstituteLjubljana, Slovenia

**Keywords:** amyloid pore, amyloid toxicity, toxic oligomers, cystatin B, lipid membranes

## Abstract

It is well documented that amyloid forming peptides and proteins interact with membranes and that this correlates with cytotoxicity. To introduce the theme we give a brief description of some amyloidogenic proteins and note their similarities with pore forming toxins (PFTs) and cell penetrating peptides. Human stefin B, a member of the family of cystatins, is an amyloidogenic protein *in vitro.* This review describes our studies of the interaction of stefin B oligomers and prefibrillar aggregates with model membranes leading to pore formation. We have studied the interaction between human stefin B and artificial membranes of various compositions. We also have prepared distinct sizes and morphologies of stefin B prefibrillar states and assessed their toxicity. Furthermore, we have measured electrical currents through pores formed by stefin B prefibrillar oligomers in a planar lipid bilayer setup. We finally discuss the possible functional and pathological significance of such pores formed by human stefin B.

## Introduction

In nearly all neurodegenerative diseases, including Alzheimer's disease (AD), Parkinson's disease (PD), frontotemporal dementia, motor neuron disease, and transmissible spongiform encephalopathy (TSE), the specific disease-related protein misfolds into an alternative conformation that tends to form β-sheet rich oligomers and, eventually, amyloid fibrils. It is largely believed, and has been shown in model systems, that the prefibrillar aggregates and protofibrils are more neurotoxic than the mature and long fibrils (Stefani and Dobson, 2003; Butterfield and Lashuel, 2010). That toxicity takes place via membrane perturbation and even pore formation, is widely accepted (Stefani and Dobson, 2003; Kagan and Thundimadathil, 2010). However, the details of exactly how oligomers of amyloid forming peptides and proteins make neurons less viable, and even lead to neuronal death, remains a challenge to be resolved.

Typical amyloid forming peptides or proteins bind to membranes, regardless of specific sequence differences. These interactions involve both plasma membrane and intracellular membranes such as mitochondrial (Squier, 2001; Pagani and Eckert, 2011) and lysosomal membranes (Liu et al., 2010). The proteins undergo transitions to β-sheet rich conformations before or concomitant with interaction with membranes (Kagan and Thundimadathil, 2010). Lipids can facilitate or induce the change of protein conformations from unfolded into α-helix or β-sheet-rich structures (Butterfield and Lashuel, 2010). Many of the amyloid forming proteins perforate the membranes and form actual pores (Kagan and Thundimadathil, 2010).

Morphologically and structurally, the amyloid pores are similar to those formed by other pore-forming proteins (Parker and Feil, 2005; Anderluh and Lakey, 2008). They have been detected in around 20 amyloid forming proteins (some are listed in Table 1), ranging from typical globular to intrinsically disordered proteins or proteolytic fragments of amyloidogenic proteins. The pores are in general quite large (3–10 nm in diameter) and relatively non-selective for ion traffic (Butterfield and Lashuel, 2010; Kagan and Thundimadathil, 2010). Well defined sizes and morphologies have been reported for some amyloid pores such as those from α-synuclein (Lashuel et al., 2002) or Aβ peptide (Quist et al., 2005).

**Table 1:** **Some cases of amyloid and pore forming proteins and peptides [compiled from Tables 1 and 2 in Kagan and Thundimadathil (2010)]**.

**Disease**	**Protein, pores shown *in vitro***	**References**
Alzheimer's disease (AD)	Amyloid precursor protein, Aβ peptides (1–40, 1–42)	Demuro et al., 2011; Kawahara et al., 2011
Familial amyloid polyneuropathy (finish)	Gelsolin	Kagan et al., 2012
Familial amyloid polyneuropathy	Transthyretin	Hirakura et al., 2001
Hereditary amyloid angiopathy	Cystatin C	Kagan and Thundimadathil, 2010
Dialysis associated amyloidosis	β-2-microglobulin	Hirakura and Kagan, 2001
Parkinson's disease (PD)	α-synuclein, NAC (α-synuclein fragment 65–95)	Lashuel et al., 2002
Variant Creutzfeldt–Jakob disease	Prion protein	Kagan and Thundimadathil, 2010
Non-amyloid neurodegenerative disease (EPM1)	Human stefin B (cystatin B)	Rabzelj et al., 2008
Type II diabetes mellitus	Islet amyloid polypeptide IAPP (amylin)	Mirzabekov et al., 1996

In this review we describe membrane interactions and pore formation induced by human stefin B, some of its mutants and different oligomeric states. We also discuss what is known about the possible physiological consequences. The pore forming activities of some other amyloidogenic proteins and peptides are considered and compared to the well-known pore forming proteins.

## Protein folding and oligomerization are modulated by membranes

Proteins fold as encoded in their primary sequence (Anfinsen, 1973). However, in the cell, protein folding takes a regulated route (Jaikaran et al., 2004). It takes place in a very crowded milieu, often encountering hydrophobic membrane surfaces. The influence of membranes on protein folding and unfolding is manifold. Important factors are membrane composition and micro-domain structure. At membranes, proteins are at higher local concentrations, the pH is lower, the dielectric constant drops from 80 in water to about 2 in hydrophobic environments (Torres-Bugeau et al., 2011). This all influences the conformation of proteins.

Ordered microdomains and less ordered lipid rafts both participate in amyloid-induced neurotoxicity. The ordered microdomains are composed of glycosphingolipids, cholesterol, and sphingomyelin. Although difficult to probe, in living cells plasma membranes co-exist as liquid crystalline and gel-like domains (Mamdouh et al., 1998). Microdomains are part of amyloid plaques of several origins (Gellermann et al., 2005), supporting the suggestion that the oligomers of amyloid-forming proteins assemble on microdomains (Zampagni et al., 2010).

It also is generally recognized that amyloid oligomers preferentially bind lipids with anionic character, regardless of their charge state (Torres-Bugeau et al., 2011).

## Pore forming proteins are part of innate immunity

Pore forming proteins are able to perforate lipid membranes (Anderluh and Lakey, 2010). Pore formation may be part of a normal physiological mechanism or used by organisms for preying or defence (Iacovache et al., 2008; Feil et al., 2010). Pore formation in lipid membranes is an ancient way of attack by various organisms. Typical examples are the bacterial pore forming toxins (PFTs) that are important virulence factors (Anderluh and Lakey, 2008). They damage eukaryotic cell membranes and help in spreading the bacteria within its host (Tweten, 2005; Bischofberger et al., 2009).

Further, pore forming proteins of the mammalian immune system, such as perforin or the membrane attack complex of complement, are designed to remove unwanted cells from the body (Voskoboinik et al., 2006; Rosado et al., 2008). Antimicrobial host defence peptides (Kagan et al., 2012), such as defensins and protegrins, are part of innate immunity. These predominantly β-sheet structure adopting peptides are toxic to bacteria, fungi, and viruses. Jang et al. (2011), demonstrated that the 18 amino acid long protegrin-1 forms amyloid-like fibrils. The action of protegrins, when released from the granules residing in macrophages and neutrophils, is mediated by channel formation (Sokolov et al., 1999; Capone et al., 2010). Temporins, peptides of up to 14 amino-acids and initially in the conformation of an amphipathic α-helix, have selective lipid binding properties and discriminate host from target cells. In innate immunity, host defence peptides target membranes of cancerous cells rather than of healthy cells (Riedl et al., 2011), due to anionic lipid phosphatidyl serine exposed on the outer membrane of cancer cells. Another source of negative charges on cells are sialic acid residues, part of the glycosylation of proteins and lipids, and heparan and chondroitin sulphate groups of proteoglycans.

Over the years many structural and functional studies have provided an insight into the mechanism of pore formation and the architecture of transmembrane pores. The pores are composed of either transmembrane β-barrels (Heuck et al., 2001) or clusters of α-helices (Kristan et al., 2009; Kagan and Thundimadathil, 2010). The sizes of the final pores vary and range from 2 up to 40 nm in diameter (Bischofberger et al., 2009). The pores allow uncontrolled flow of ions and small molecules, or even proteins in the case of large pores. The consequences of pore formation differ and depend on the number of pores present in the plasma membrane, the mechanism of membrane binding, cell type, etc.

Apart from cell plasma membranes the intracellular lysosomal membrane can also be the target of pore forming proteins. Some bacteria can hide in a latent state in endosomes until they eventually exit the cell by autophagy (Bischofberger et al., 2009). This again resembles the sequestration of amyloid aggregates, such as, aggresomes and their removal from the cell by autophagy (Perlmutter, 2006; Liu et al., 2010). One additional similarity between amyloid oligomers and pore forming proteins is a multistep mechanism of channel formation, which includes oligomerization in the plane of the membrane (Bischofberger et al., 2009). “Annular” oligomers of amyloid forming proteins thus resemble the pores of pore forming proteins.

## Pores by amyloid forming proteins

Transmembrane pores of amyloidogenic proteins are believed to start to form from ring-like structures, i.e., annular oligomers and resembling β barrels of pore-forming toxins. However, the conformation of prefibrillar species is not always composed of β-structure. There are cases where temporarily non-native α-helical structure forms that, in contact with the membrane, transforms to a β-structure arrangement, and again resembling some of the PFTs (Kagan and Thundimadathil, 2010).

As a prototype of such pores, amyloid-β (Aβ) peptide has been studied. Aβ in its soluble and oligomeric form causes synaptic damage in AD (Walsh et al., 2002) and problems with memory (Lesne et al., 2006). Aβ (1–40) adopts a mixture of secondary structures in solution. A lipid surface induces change in the conformation and, after insertion, the peptide adopts an all-β conformation (Williams and Serpell, 2011). It has been shown that (Aβ) oligomers of different sizes, which also bind Congo red, incorporate into lipid membranes and permeabilize them (Williams et al., 2010; Williams and Serpell, 2011).

Arispe et al. (1993), reported that amyloid-β (Aβ) forms ion-channels in lipid bilayer membranes. The channels, nowadays termed pores, were rather large, non-selective for various cations, Ca^2+^ permeable and could be blocked by Zn^2+^ (Arispe et al., 1996; Kawahara et al., 1997).

It has been widely speculated that the pore forming ability of amyloid peptides may cause imbalance of ions in the cell. Thus, binding of toxic amyloid oligomers to the plasma membrane and pore formation would increase influx of Ca^2+^, while pore formation in mitochondrial or lysosomal membranes would further contribute to reactive oxidative species (ROS) and metabolic imbalance, finally leading to apoptosis (Bucciantini et al., 2002; Stefani and Dobson, 2003; Stefani, 2010).

In the case of (Aβ) this hypothesis has been confirmed in real brain slices. Kawahara et al. (2011) showed that oligomeric Aβ incorporates directly into neural membranes and forms ion channels. The channels are cation-selective and lead to disruption of calcium homeostasis. A study by Demuro (Demuro et al., 2011) has confirmed that “single-channel Ca^2+^ imaging implicates Aβ (1–42) amyloid pores in AD pathology.”

In due course, many other amyloid peptides and proteins, among them human islet amylin, prion protein, and α-synuclein (Kawahara et al., 2000; Lashuel et al., 2002), have been shown to form pores in membranes and consequently to increase intracellular Ca^2+^.

α-Synuclein is a protein, mutations of which have been shown to lead to protein accumulation in Lewy bodies, with a direct link to PD. It is known that the N-terminal part of α-synuclein is the membrane binding region (Butterfield and Lashuel, 2010). Molecular dynamics simulations suggest that it adopts an α 11/3 helix (Mihajlovic and Lazaridis, 2008). If this part is deleted, α-synuclein is no longer toxic to yeast (Volles and Lansbury, 2007; Vamvaca et al., 2009). α-Synuclein, like many amyloid forming proteins, binds preferentially to anionic membranes and the dimeric form binds more strongly to lipid membranes than the monomer (Giannakis et al., 2008). Protofibrillar α-synuclein was shown to permeabilize lipid membranes and exerts a “pore-like mechanism” (Volles and Lansbury, 2002).

Human amylin is a polypeptide of 37-amino acids secreted from pancreatic islet β-cells. It accumulates as islet amyloid in type 2 diabetes. Human amylin (islet amyloid polypeptide—IAPP) disrupts membrane integrity (Last et al., 2011) and readily forms ion pores in planar lipid bilayers (Mirzabekov et al., 1996). Initially it binds to anionic vesicles in an α-helical conformation, where the amphipathic α-helix binds in parallel to the membrane surface. Factors of membrane microenvironment and protein concentration transform the peptide to a β-sheet conformation and amyloid-like aggregates (Butterfield and Lashuel, 2010).

Not only intrinsically disordered proteins such as α-synuclein and prion protein, or largely unstructured peptides such as amylin or Aβ, were shown to form pores. Even globular proteins such as β2-microglobulin (Hirakura and Kagan, 2001) or stefin B (described in this review—as below) have similar properties, when they undergo conversion to a β-rich conformation and form prefibrillar oligomers.

An interesting example is BASP1, an abundant brain protein, which is myristoylated and situated on the inner side of the presynaptic plasma membrane. Until now, this protein has not been implicated in any amyloid disease. However, electrophysiological recordings have demonstrated that BASP1 channels are similar to amyloid protein channels. They induce single channel currents and independent of the voltage, into negatively charged planar lipid bilayers (Ostroumova et al., 2011).

In Table 1 some examples of the amyloid proteins, which have been proven to make pores, are listed; a more extensive number has been described elsewhere (Anderluh and Lakey, 2010; Kagan and Thundimadathil, 2010). Comprehensive reviews of the ability of amyloid proteins to form pores have been written by Kagan and colleagues (Kagan et al., 2004, 2012; Kagan and Thundimadathil, 2010).

## Pore formation by human stefin B

Human stefin B (cystatin B) is a cysteine protease inhibitor (Turk et al., 2008). Its mutants cause a rare progressive myoclonus epilepsy of type 1—EPM1 (Lalioti et al., 1997; Genton, 2010). Stefin B is a small globular protein of 11 kDa with no disulphide bonds. This intracellular protein resides both in the cytoplasm and in the nucleus, where it was shown to bind to histones, however, its exact nuclear function is not yet known (Ceru et al., 2010). It may have alternative functions, for example it was implicated in innate immunity and NO production (Lefebvre et al., 2004) or prevention of oxidative stress (Lehtinen et al., 2009). It was found to form oligomers *in vitro* and in the cellular environment (Cipollini et al., 2008). It also was proven to be amyloidogenic *in vitro* (Zerovnik et al., 2002a, 2007). Its homolog, human stefin A, similarly forms amyloid fibrils and dimers *in vitro*, however, this happens only under extreme conditions of temperature and pH (Jenko et al., 2004). Many amyloid forming proteins, especially in prefibrillar and oligomeric states, have been shown to bind to membranes and to perforate them (Kawahara et al., 2000). For these reasons, stefin B membrane interactions and cyto-toxicity have been studied in detail.

**Figure 1:**
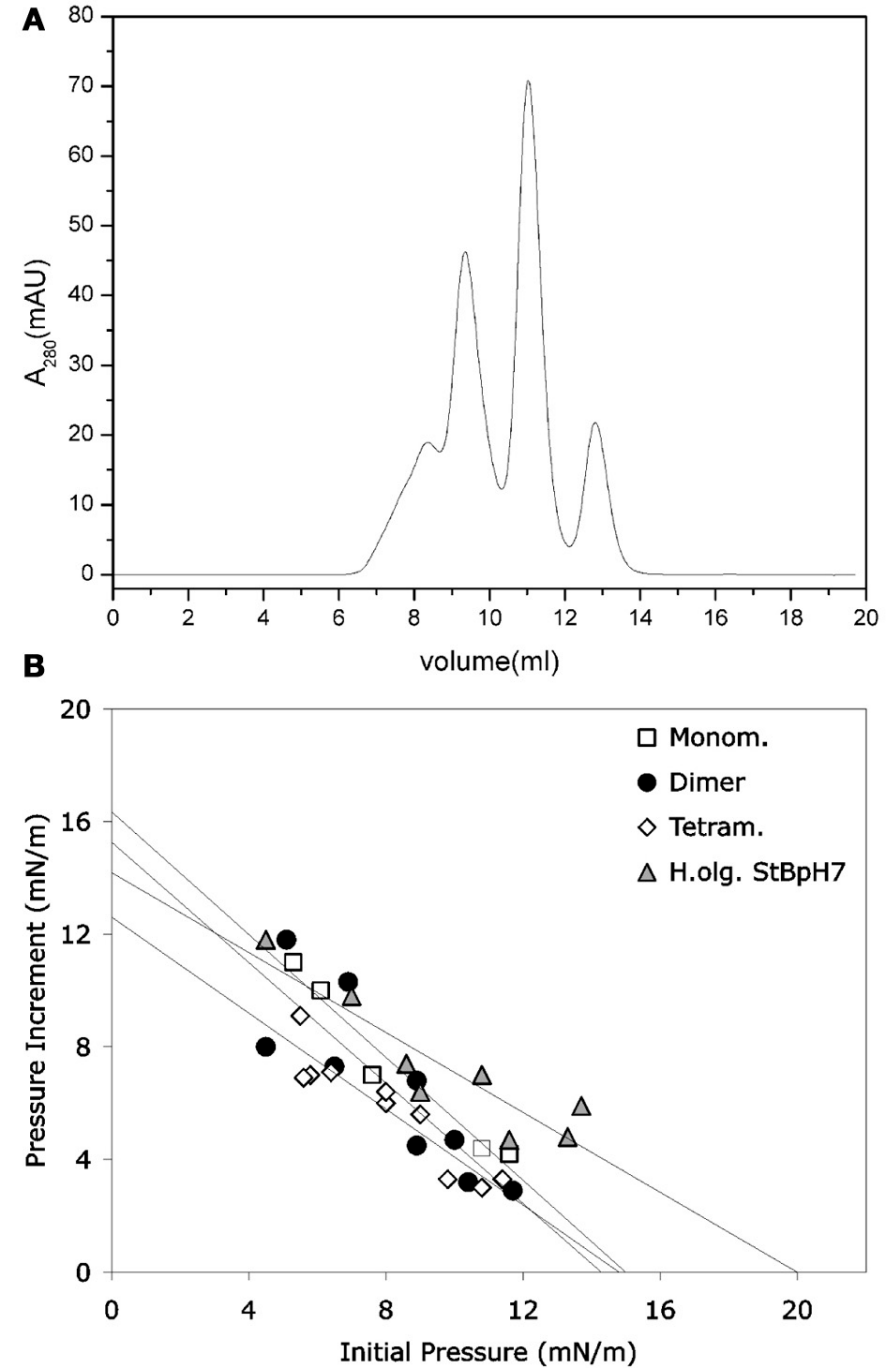
**Monomers and separated oligomers of human recombinant stefin B-E31 variant. (A)** Elution volumes of a sample composed of monomers, dimers, tetramers, and higher oligomers after application to SEC Superdex 75 column. **(B)** Only higher oligomers (triangles) were shown to insert into lipid monolayers to some degree. **(B)** modified from Amyloid, Informa Health Care with permission.

It was shown that stefin B forms amyloid fibrils *in vitro* under mild, physiologically relevant conditions (Zerovnik et al., 2002a,b,c), unlike the more stable homologous human stefin A (Jenko et al., 2004). The existence of different prefibrillar species at pH 3, high salt (starting from a structured molten globule and bearing helical structure), or at pH 5, 0.15 M salt (starting from a native-like intermediate) was shown. In both cases, after some time, all-β sheet structure formed, concomitant with fibril formation (Zerovnik et al., 2002b). Oligomerization to globules of sizes from 5 to 10 nm at pH 5 and from 15 to 20 nm (cross-diameter) at pH 3 was observed for the prefibrillar states, respectively. These oligomers contained from 6 to 32 monomers, as judged by the approximate volume of the molecules and confirmed by size exclusion chromatography and dynamic light scattering (Jenko Kokalj et al., 2007; Ceru et al., 2008). Therefore, well-defined oligomers of stefin B may be prepared by size exclusion chromatography (Figure 1A) (Ceru et al., 2008), in contrast to some other systems such as Aβ or α-synuclein.

The prefibrillar states were prepared at pH 4.8 and 3.3, as described (Zerovnik et al., 2002b; Ceru et al., 2008). The interaction of the prefibrillar oligomers and aggregates of stefin B with membranes was studied by various biophysical approaches. In our first paper on the interaction of the prefibrillar oligomeric forms of stefin B with membranes and correlation with toxicity by Anderluh et al. (2005), we showed that toxicity to SH-SY5Y neuroblastoma cells was produced by the prefibrillar aggregates of stefin B, obtained at both pH 4.8 and 3.3, and was concentration dependent. However, even the protein at pH 7.3 was slightly toxic. This can be understood as it is actually a mixture of oligomers (Rabzelj et al., 2005; Ceru et al., 2008). The prefibrillar states of stefin B showed concentration-dependent release of the fluorescent probe calcein from unilamellar lipid vesicles composed of negatively charged lipids. Liposomes composed solely of phosphatidylcholine were not permeabilized. A homologous protein, stefin A, did not permeabilize liposomes of any lipid composition. Membrane interactions were further studied by surface plasmon resonance (SPR) and lipid monolayer insertion. In both cases prefibrillar aggregates showed interactions predominantly with negatively charged lipids (Anderluh et al., 2005).

**Figure 2:**
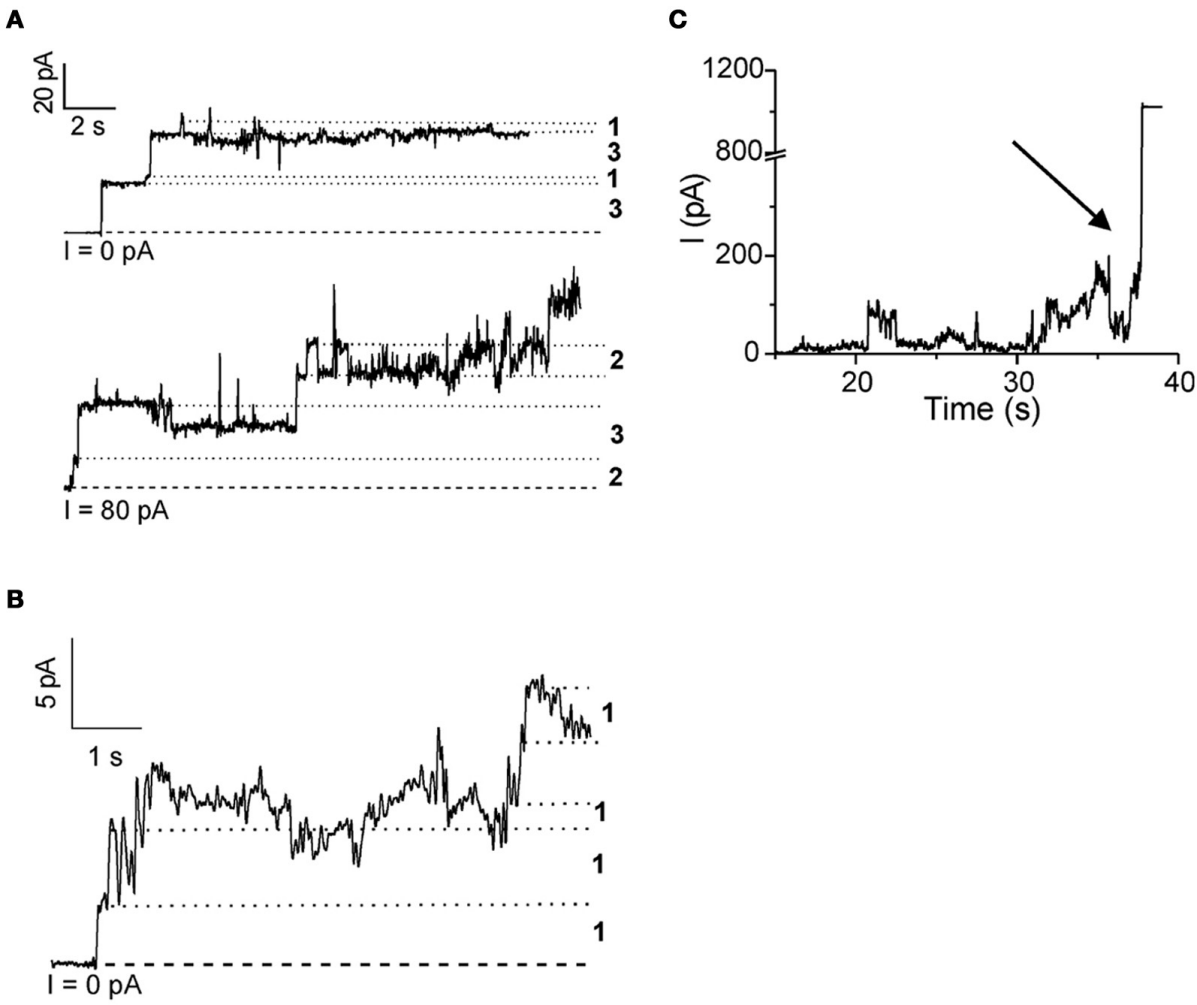
**Pore formation in planar lipid bilayers by some of the stefin B variants.** Pore formation measured by electrophysiological recordings is shown for native wild-type stefin B **(A)** stefin B-Y31 variant **(B)** and membrane destabilization by G4R **(C)**. The break of the membrane is denoted by an arrow. Figure modified from *FEBS J*., John Wiley and Sons with permission.

Rabzelj et al. (2008) have shown that wild-type (wt) stefin B and its variant with tyrosine at position 31 (stefin B-Y31), instead of the glutamic acid in the wt, can form pores in the planar lipid bilayers setup (Figure 2). A mutant observed in patients with EPM1—G4R of the wt protein was also studied. This mutant is stable and folded (Rabzelj et al., 2005) albeit it differs in one positive charge from the wt. In the case of wt protein discrete increases of the current were observed, indicating pore opening (Figure 2) while, with the prefibrillar state of stefin B-Y31 variant, larger pores were observed that remained open for longer times. The G4R mutant exhibited very rapid and stochastic membrane damaging effects. It was frequently observed that addition of G4R led to membrane breaks after some minutes, indicating very strong membrane interaction (Figure 2). These results were in agreement with SPR measurements and insertion into lipid monolayers (Rabzelj et al., 2008).

Not only the acid aggregated states of stefin B but also the higher oligomers (higher than tetramers) of the wild type (wt) protein isolated at pH 7 proved toxic to cells (Ceru et al., 2008). What is of interest here is the clear distinction between oligomer size and toxicity. In the case of stefin B wt, the monomers, dimers, and tetramers are not toxic and toxicity starts with the higher species (Figure 1A) such as hexamers and dodecamers. As known from studies by TEM and AMF, such structures resemble micelles and are annular in shape, which is the case for membrane interacting oligomers of other amyloid-forming toxins, such as α-synuclein (Lashuel et al., 2002). The oligomers from 6 to 12-mers inserted better than monomers, dimers, and tetramers (Figure 1B). The lipid monolayer insertion correlated well with the cytotoxicity of isolated oligomers (Ceru et al., 2008).

## Possible functional role of the oligomers and pores of stefin B

Amyloid fibrils, from bacteria to humans, can serve a functional physiological role under certain conditions (Fowler et al., 2007). Such is the well-known example of polymeric and fibrillar Pmel17 which plays an important role in the biosynthesis and storage of the pigment melanin. Intracellular Pmel17 amyloidogenesis is carefully orchestrated by the secretory pathway, utilizing membrane sequestration and proteolysis to protect the cell from potentially toxic amyloidogenic intermediates (Fowler et al., 2006; Watt et al., 2009). Recently, prion-like amplification of oligomers by *Drosophila* Orb2 was implicated in long-term memory (Majumdar et al., 2012) as predicted earlier (Wickelgren, 2004).

In our study of membrane interaction and pore formation by stefin B native state and its oligomers, including prefibrillar aggregates (Anderluh et al., 2005; Rabzelj et al., 2008), we have shown that the wild type protein forms cation selective pores (Rabzelj et al., 2008), which is not necessarily deleterious for the cell. It was suggested that pores formed by Aβ may be prosurvival (Plant et al., 2006).

Stefin B oligomers do exist in cells (Cipollini et al., 2008). However, at present there is no evidence, as was demonstrated for Aβ (Kawahara et al., 2011) in AD, that pores formed by stefin B *in vitro* also form in cells. Regarding their potential functional role, this protein has some additional, cathepsin independent functions. It forms part of the cytoskeletal complex (Di Giaimo et al., 2002), it may be part of cellular signaling to the nucleus, where it was found to bind histones (Ceru et al., 2010), and it has an anti-oxidant function (Lehtinen et al., 2009). Its protective, anti-oxidant and anti-aging roles may be similar to those of cystatin C, which was in addition shown to induces autophagy (Tizon et al., 2010a,b) by an as yet unknown mechanism. We propose that some of these alternative functions may be due to the protein's oligomeric states. It has been reported that stefin B is involved in the invertebrate innate immunity response (Lefebvre et al., 2004), a precursor to the mammalian system (Salzet et al., 2006). In accordance with their neuro-protective role, stefin B is overexpressed after seizures (D'Amato et al., 2000) and cystatin C in status epilepticus (Pirttila et al., 2005; Kaur et al., 2010).

## Conclusions

Amyloid forming proteins have a strong tendency to interact with lipid membranes and to make pores (perforate the membranes). The behavior of amyloid forming proteins is, in many ways, comparable to that of bacterial toxins and innate immunity membrane perforating proteins, as well as anti-bacterial peptides.

Stefin B prefibrillar states, as well as higher oligomers, hexamers and higher, behave similarly to those of other amyloid forming proteins and peptides. What is of interest in the case of stefin B is the observation that the wild type protein also forms smaller pores as well as the rather stable G4R mutant observed in some patients with EPM1 (Rabzelj S, 2006, PhD thesis, University of Ljubljana). This latter mutant exhibits especially strong perturbation on membranes. We believe that pore formation by the wild type stefin B may be related to epileptogenesis, either by exerting some protective/regulatory function on the membrane excitability, or by acting via the host defence system. It is known that stefin B is involved in the innate immunity response (Lefebvre et al., 2004) and that it is over-expressed after epileptic seizures (D'Amato et al., 2000).

### Conflict of interest statement

The authors declare that the research was conducted in the absence of any commercial or financial relationships that could be construed as a potential conflict of interest.

## References

[B1] Anderluh G. Gutierrez-Aguirre I. Rabzelj S. Ceru S. Kopitar-Jerala N. Macek P. Turk V. Zerovnik E. (2005). Interaction of human stefin B in the prefibrillar oligomeric form with membranes. Correlation with cellular toxicity. FEBS J. 272, 3042–3051 10.1111/j.1742-4658.2005.04717.x 15955063

[B2] Anderluh G. Lakey J. (2010). Proteins: membrane binding and pore formation. Preface. Adv. Exp. Med. Biol. 677, v–vi 20687475

[B3] Anderluh G. Lakey J. H. (2008). Disparate proteins use similar architectures to damage membranes. Trends Biochem. Sci. 33, 482–490 10.1016/j.tibs.2008.07.004 18778941

[B4] Anfinsen C. B. (1973). Principles that govern the folding of protein chains. Science 181, 223–230 10.1126/science.181.4096.223 4124164

[B5] Arispe N. Pollard H. B. Rojas E. (1996). Zn2+ interaction with Alzheimer amyloid beta protein calcium channels. Proc. Natl. Acad. Sci. U.S.A. 93, 1710–1715 8643694 10.1073/pnas.93.4.1710 PMC40007

[B6] Arispe N. Rojas E. Pollard H. B. (1993). Alzheimer disease amyloid beta protein forms calcium channels in bilayer membranes: blockade by tromethamine and aluminum. Proc. Natl. Acad. Sci. U.S.A. 90, 567–571 8380642 10.1073/pnas.90.2.567 PMC45704

[B7] Bischofberger M. Gonzalez M. R. van der Goot F. G. (2009). Membrane injury by pore-forming proteins. Curr. Opin. Cell Biol. 21, 589–595 10.1016/j.ceb.2009.04.003 19442503

[B8] Bucciantini M. Giannoni E. Chiti F. Baroni F. Formigli L. Zurdo J. Taddei N. Ramponi G. Dobson C. M. Stefani M. (2002). Inherent toxicity of aggregates implies a common mechanism for protein misfolding diseases. Nature 416, 507–511 10.1038/416507a 11932737

[B9] Butterfield S. M. Lashuel H. A. (2010). Amyloidogenic protein membrane interactions: mechanistic insight from model systems. Angew. Chem. Int. Ed. 49, 5628–5654 10.1002/anie.200906670 20623810

[B10] Capone R. Mustata M. Jang H. Arce F. T. Nussinov R. Lal R. (2010). Antimicrobial protegrin-1 forms ion channels: molecular dynamic simulation, atomic force microscopy, and electrical conductance studies. Biophys. J. 98, 2644–2652 10.1016/j.bpj.2010.02.024 20513409 PMC2877344

[B11] Ceru S. Kokalj S. J. Rabzelj S. Skarabot M. Gutierrez-Aguirre I. Kopitar-Jerala N. Anderluh G. Turk D. Turk V. Zerovnik E. (2008). Size and morphology of toxic oligomers of amyloidogenic proteins: a case study of human stefin B. Amyloid 15, 147–159 10.1080/13506120802193555 18925453

[B12] Ceru S. Konjar S. Maher K. Repnik U. Krizaj I. Bencina M. Renko M. Nepveu A. Zerovnik E. Turk B. Kopitar-Jerala N. (2010). Stefin B interacts with histones and cathepsin L in the nucleus. J. Biol. Chem. 285, 10078–10086 10.1074/jbc.M109.034793 20075068 PMC2843170

[B13] Cipollini E. Riccio M. Di Giaimo R. Dal Piaz F. Pulice G. Catania S. Caldarelli I. Dembic M. Santi S. Melli M. (2008). Cystatin B and its EPM1 mutants are polymeric and aggregate prone *in vivo*. Biochim. Biophys. Acta 1783, 312–322 10.1016/j.bbamcr.2007.08.007 17920138

[B14] D'Amato E. Kokaia Z. Nanobashvili A. Reeben M. Lehesjoki A. E. Saarma M. Lindvall O. (2000). Seizures induce widespread upregulation of cystatin B, the gene mutated in progressive myoclonus epilepsy, in rat forebrain neurons. Eur. J. Neurosci. 12, 1687–1695 10.1046/j.1460-9568.2000.00058.x 10792446

[B15] Demuro A. Smith M. Parker I. (2011). Single-channel Ca(2+) imaging implicates Abeta1-42 amyloid pores in Alzheimer's disease pathology. J. Cell Biol. 195, 515–524 10.1083/jcb.201104133 22024165 PMC3206345

[B16] Di Giaimo R. Riccio M. Santi S. Galeotti C. Ambrosetti D. C. Melli M. (2002). New insights into the molecular basis of progressive myoclonus epilepsy: a multiprotein complex with cystatin B. Hum. Mol. Genet. 11, 2941–2950 10.1093/hmg/11.23.2941 12393805

[B17] Feil S. C. Polekhina G. Gorman M. A. Parker M. W. (2010). Proteins membrane binding and pore formation introduction. Adv. Exp. Med. Biol. 677, 1–13 20687476

[B18] Fowler D. M. Koulov A. V. Alory-Jost C. Marks M. S. Balch W. E. Kelly J. W. (2006). Functional amyloid formation within mammalian tissue. PLoS Biol. 4:e6 10.1371/journal.pbio.0040006 16300414 PMC1288039

[B19] Fowler D. M. Koulov A. V. Balch W. E. Kelly J. W. (2007). Functional amyloid–from bacteria to humans. Trends Biochem. Sci. 32, 217–224 10.1016/j.tibs.2007.03.003 17412596

[B20] Gellermann G. P. Appel T. R. Tannert A. Radestock A. Hortschansky P. Schroeckh V. Leisner C. Lutkepohl T. Shtrasburg S. Rocken C. Pras M. Linke R. P. Diekmann S. Fandrich M. (2005). Raft lipids as common components of human extracellular amyloid fibrils. Proc. Natl. Acad. Sci. U.S.A. 102, 6297–6302 10.1073/pnas.0407035102 15851687 PMC1088351

[B21] Genton P. (2010). Unverricht-Lundborg disease (EPM1). Epilepsia 51, 37–39 10.1111/j.1528-1167.2009.02441.x 20331711

[B22] Giannakis E. Pacifico J. Smith D. P. Hung L. W. Masters C. L. Cappai R. Wade J. D. Barnhain K. J. (2008). Dimeric structures of alpha-synuclein bind preferentially to lipid membranes. Biochim. Biophys. Acta 1778, 1112–1119 10.1016/j.bbamem.2008.01.012 18261456

[B23] Heuck A. P. Tweten R. K. Johnson A. E. (2001). Beta-barrel pore-forming toxins: intriguing dimorphic proteins. Biochemistry 40, 9065–9073 11478872 10.1021/bi0155394

[B24] Hirakura Y. Azimova R. Azimov R. Kagan B. (2001). Ion channels with different selectivity formed by TTR. Biophys. J. 80, 129a

[B25] Hirakura Y. Kagan B. L. (2001). Pore formation by beta-2-microglobulin: a mechanism for the pathogenesis of dialysis associated amyloidosis. Amyloid 8, 94–100 11409039 10.3109/13506120109007350

[B26] Iacovache I. van der Goot F. G. Pernot L. (2008). Pore formation: an ancient yet complex form of attack. Biochim. Biophys. Acta 1778, 1611–1623 10.1016/j.bbamem.2008.01.026 18298943

[B27] Jaikaran E. T. A. S. Nilsson M. R. Clark A. (2004). Pancreatic beta-cell granule peptides form heteromolecular complexes which inhibit islet amyloid polypeptide fibril formation. Biochem. J. 377, 709–716 10.1042/BJ20030852 14565847 PMC1223903

[B28] Jang H. Arce F. T. Mustata M. Ramachandran S. Capone R. Nussinov R. Lal R. (2011). Antimicrobial protegrin-1 forms amyloid-like fibrils with rapid kinetics suggesting a functional link. Biophys. J. 100, 1775–1783 10.1016/j.bpj.2011.01.072 21463591 PMC3072611

[B29] Jenko S. Skarabot M. Kenig M. Guncar G. Musevic I. Turk D. Zerovnik E. (2004). Different propensity to form amyloid fibrils by two homologous proteins-Human stefins A and B: searching for an explanation. Proteins 55, 417–425 10.1002/prot.20041 15048832

[B30] Jenko Kokalj S. Guncar G. Stern I. Morgan G. Rabzelj S. Kenig M. Staniforth R. A. Waltho J. P. Zerovnik E. Turk D. (2007). Essential role of proline isomerization in stefin B tetramer formation. J. Mol. Biol. 366, 1569–1579 10.1016/j.jmb.2006.12.025 17217964

[B31] Kagan B. L. Azimov R. Azimova R. (2004). Amyloid peptide channels. J. Membr. Biol. 202, 1–10 10.1007/s00232-004-0709-4 15702375

[B33] Kagan B. L. Jang H. Capone R. Teran Arce F. Ramachandran S. Lal R. Nussinov R. (2012). Antimicrobial properties of amyloid peptides. Mol. Pharm. 9, 708–717 10.1021/mp200419b 22081976 PMC3297685

[B34] Kagan B. L. Thundimadathil J. (2010). Amyloid peptide pores and the beta sheet conformation. Adv. Exp. Med. Biol. 677, 150–167 20687488 10.1007/978-1-4419-6327-7_13

[B37a] Kaur G. Mohan P. Pawlik M. DeRosa S. Fajiculay J. Che S. L. Grubb A. Ginsberg S. D. Nixon R. A. Levy E. (2010). Cystatin C rescues degenerating neurons in a cystatin B-knockout mouse model of progressive myoclonus epilepsy. Am. J. Pathol. 177, 2256–2267 10.2353/ajpath.2010.100461 20889561 PMC2966785

[B35] Kawahara M. Arispe N. Kuroda Y. Rojas E. (1997). Alzheimer's disease amyloid beta-protein forms Zn(2+)-sensitive, cation-selective channels across excised membrane patches from hypothalamic neurons. Biophys. J. 73, 67–75 10.1016/S0006-3495(97)78048-2 9199772 PMC1180909

[B36] Kawahara M. Kuroda Y. Arispe N. Rojas E. (2000). Alzheimer's beta-amyloid, human islet amylin, and prion protein fragment evoke intracellular free calcium elevations by a common mechanism in a hypothalamic GnRH neuronal cell line. J. Biol. Chem. 275, 14077–14083 10.1074/jbc.275.19.14077 10799482

[B37] Kawahara M. Ohtsuka I. Yokoyama S. Kato-Negishi M. Sadakane Y. (2011). Membrane incorporation, channel formation, and disruption of calcium homeostasis by Alzheimer's beta-amyloid protein. Int. J. Alzheimers Dis. 2011, 304583 10.4061/2011/304583 21547225 PMC3087492

[B38] Kristan K. C. Viero G. Dalla Serra M. Macek P. Anderluh G. (2009). Molecular mechanism of pore formation by actinoporins. Toxicon 54, 1125–1134 10.1016/j.toxicon.2009.02.026 19268680

[B39] Lalioti M. D. Mirotsou M. Buresi C. Peitsch M. C. Rossier C. Ouazzani R. Baldy-Moulinier M. Bottani A. Malafosse A. Antonarakis S. E. (1997). Identification of mutations in cystatin B, the gene responsible for the Unverricht-Lundborg type of progressive myoclonus epilepsy (EPM1). Am. J. Hum. Genet. 60, 342–351 9012407 PMC1712389

[B40] Lashuel H. A. Hartley D. Petre B. M. Walz T. Lansbury P. T. Jr. (2002). Neurodegenerative disease: amyloid pores from pathogenic mutations. Nature 418, 291 10.1038/418291a 12124613

[B41] Last N. B. Rhoades E. Miranker A. D. (2011). Islet amyloid polypeptide demonstrates a persistent capacity to disrupt membrane integrity. Proc. Natl. Acad. Sci. U.S.A. 108, 9460–9465 10.1073/pnas.1102356108 21606325 PMC3111278

[B42] Lefebvre C. Cocquerelle C. Vandenbulcke F. Hot D. Huot L. Lemoine Y. Salzet M. (2004). Transcriptomic analysis in the leech Theromyzon tessulatum: involvement of cystatin B in innate immunity. Biochem. J. 380, 617–625 10.1042/BJ20040478 15089746 PMC1224237

[B43] Lehtinen M. K. Tegelberg S. Schipper H. Su H. Zukor H. Manninen O. Kopra O. Joensuu T. Hakala P. Bonni A. Lehesjoki A. E. (2009). Cystatin B deficiency sensitizes neurons to oxidative stress in progressive myoclonus epilepsy, EPM1. J. Neurosci. 29, 5910–5915 10.1523/JNEUROSCI.0682-09.2009 19420257 PMC2694495

[B44] Lesne S. Koh M. T. Kotilinek L. Kayed R. Glabe C. G. Yang A. Gallagher M. Ashe K. H. (2006). A specific amyloid-beta protein assembly in the brain impairs memory. Nature 440, 352–357 10.1038/nature04533 16541076

[B45] Liu C. Gao Y. Barrett J. Hu B. (2010). Autophagy and protein aggregation after brain ischemia. J. Neurochem. 115, 68–78 10.1111/j.1471-4159.2010.06905.x 20633207 PMC3518272

[B46] Liu R. Q. Zhou Q. H. Ji S. R. Zhou Q. Feng D. Wu Y. Sui S. F. (2010). Membrane localization of beta-amyloid 1-42 in lysosomes: a possible mechanism for lysosome labilization. J. Biol. Chem. 285, 19986–19996 10.1074/jbc.M109.036798 20430896 PMC2888410

[B47] Majumdar A. Cesario W. C. White-Grindley E. Jiang H. Ren F. Khan M. R. Li L. Choi E. M. Kannan K. Guo F. Unruh J. Slaughter B. Si K. (2012). Critical role of amyloid-like oligomers of Drosophila Orb2 in the persistence of memory. Cell 148, 515–529 10.1016/j.cell.2012.01.004 22284910

[B48] Mamdouh Z. Giocondi M. C. Le Grimellec C. (1998). In situ determination of intracellular membrane physical state heterogeneity in renal epithelial cells using fluorescence ratio microscopy. Eur. Biophys. J. 27, 341–351 9691463 10.1007/s002490050141

[B49] Mihajlovic M. Lazaridis T. (2008). Membrane-bound structure and energetics of alpha-synuclein. Proteins 70, 761–778 10.1002/prot.21558 17729279

[B50] Mirzabekov T. A. Lin M. C. Kagan B. L. (1996). Pore formation by the cytotoxic islet amyloid peptide amylin. J. Biol. Chem. 271, 1988–1992 10.1074/jbc.271.4.1988 8567648

[B51] Ostroumova O. S. Schagina L. V. Mosevitsky M. I. Zakharov V. V. (2011). Ion channel activity of brain abundant protein BASP1 in planar lipid bilayers. FEBS J. 278, 461–469 10.1111/j.1742-4658.2010.07967.x 21156029

[B52] Pagani L. Eckert A. (2011). Amyloid-Beta interaction with mitochondria. Int. J. Alzheimers Dis. 2011, 925050 10.4061/2011/925050 21461357 PMC3065051

[B53] Parker M. W. Feil S. C. (2005). Pore-forming protein toxins: from structure to function. Prog. Biophys. Mol. Biol. 88, 91–142 10.1016/j.pbiomolbio.2004.01.009 15561302

[B54] Perlmutter D. H. (2006). The role of autophagy in alpha-1-antitrypsin deficiency: a specific cellular response in genetic diseases associated with aggregation-prone proteins. Autophagy 2, 258–263 16874089 10.4161/auto.2882

[B55] Pirttila T. J. Lukasiuk K. Hakansson K. Grubb A. Abrahamson M. Pitkanen A. (2005). Cystatin C modulates neurodegeneration and neurogenesis following status epilepticus in mouse. Neurobiol. Dis. 20, 241–253 10.1016/j.nbd.2005.03.006 16242633

[B56] Plant L. D. Webster N. J. Boyle J. P. Ramsden M. Freir D. B. Peers C. Pearson H. A. (2006). Amyloid beta peptide as a physiological modulator of neuronal ‘A’-type K+ current. Neurobiol. Aging 27, 1673–1683 10.1016/j.neurobiolaging.2005.09.038 16271805

[B57] Quist A. Doudevski I. Lin H. Azimova R. Ng D. Frangione B. Kagan B. Ghiso J. Lal R. (2005). Amyloid ion channels: a common structural link for protein-misfolding disease. Proc. Natl. Acad. Sci. U.S.A. 102, 10427–10432 10.1073/pnas.0502066102 16020533 PMC1180768

[B58] Rabzelj S. Turk V. Zerovnik E. (2005). *In vitro* study of stability and amyloid-fibril formation of two mutants of human stefin B (cystatin B) occurring in patients with EPM1. Protein Sci. 14, 2713–2722 10.1110/ps.051609705 16155205 PMC2253288

[B59] Rabzelj S. Viero G. Gutierrez-Aguirre I. Turk V. Dalla Serra M. Anderluh G. Zerovnik E. (2008). Interaction with model membranes and pore formation by human stefin B: studying the native and prefibrillar states. FEBS J. 275, 2455–2466 10.1111/j.1742-4658.2008.06390.x 18397316

[B60] Riedl S. Zweytick D. Lohner K. (2011). Membrane-active host defense peptides–challenges and perspectives for the development of novel anticancer drugs. Chem. Phys. Lipids 164, 766–781 10.1016/j.chemphyslip.2011.09.004 21945565 PMC3220766

[B61] Rosado C. J. Kondos S. Bull T. E. Kuiper M. J. Law R. H. P. Buckle A. M. Voskoboinik I. Bird P. I. Trapani J. A. Whisstock J. C. Dunstone M. A. (2008). The MACPF/CDC family of pore-forming toxins. Cell. Microbiol. 10, 1765–1774 10.1111/j.1462-5822.2008.01191.x 18564372 PMC2654483

[B62] Salzet M. Tasiemski A. Cooper E. (2006). Innate immunity in lophotrochozoans: the annelids. Curr. Pharm. Des. 12, 3043–3050 16918433 10.2174/138161206777947551

[B63] Sokolov Y. Mirzabekov T. Martin D. W. Lehrer R. I. Kagan B. L. (1999). Membrane channel formation by antimicrobial protegrins. Biochim. Biophys. Acta 1420, 23–29 10.1016/S0005-2736(99)00086-3 10446287

[B64] Squier T. C. (2001). Oxidative stress and protein aggregation during biological aging. Exp. Gerontol. 36, 1539–1550 10.1016/S0531-5565(01)00139-5 11525876

[B65] Stefani M. (2010). Biochemical and biophysical features of both oligomer/fibril and cell membrane in amyloid cytotoxicity. FEBS J. 277, 4602–4613 10.1111/j.1742-4658.2010.07889.x 20977664

[B66] Stefani M. Dobson C. M. (2003). Protein aggregation and aggregate toxicity: new insights into protein folding, misfolding diseases and biological evolution. J. Mol. Med. 81, 678–699 10.1007/s00109-003-0464-5 12942175

[B67] Tizon B. Ribe E. M. Mi W. Troy C. M. Levy E. (2010a). Cystatin C protects neuronal cells from amyloid-beta-induced toxicity. J. Alzheimers Dis. 19, 885–894 10.3233/JAD-2010-1291 20157244 PMC2889175

[B68] Tizon B. Sahoo S. Yu H. Gauthier S. Kumar A. R. Mohan P. Figliola M. Pawlik M. Grubb A. Uchiyama Y. Bandyopadhyay U. Cuervo A. M. Nixon R. A. Levy E. (2010b). Induction of autophagy by cystatin C: a mechanism that protects murine primary cortical neurons and neuronal cell lines. PLoS ONE 5:e9819 10.1371/journal.pone.0009819 20352108 PMC2843718

[B69] Torres-Bugeau C. M. Borsarelli C. D. Minahk C. J. Chehin R. N. (2011). The key role of membranes in amyloid formation from a biophysical perspective. Curr. Protein Pept. Sci. 12, 166–180 21348838 10.2174/138920311795860197

[B70] Turk V. Stoka V. Turk D. (2008). Cystatins: biochemical and structural properties, and medical relevance. Front. Biosci. 13, 5406–5420 18508595 10.2741/3089

[B71] Tweten R. K. (2005). Cholesterol-dependent cytolysins, a family of versatile pore-forming toxins. Infect. Immun. 73, 6199–6209 10.1128/IAI.73.10.6199-6209.2005 16177291 PMC1230961

[B72] Vamvaca K. Volles M. J. Lansbury P. T. Jr. (2009). The first N-terminal amino acids of alpha-synuclein are essential for alpha-helical structure formation *in vitro* and membrane binding in yeast. J. Mol. Biol. 389, 413–424 10.1016/j.jmb.2009.03.021 19285989 PMC2801807

[B73] Volles M. J. Lansbury P. T. (2002). Vesicle permeabilization by protofibrillar alpha-synuclein is sensitive to Parkinson's disease-linked mutations and occurs by a pore-like mechanism. Biochemistry 41, 4595–4602 10.1021/bi0121353 11926821

[B74] Volles M. J. Lansbury P. T. Jr. (2007). Relationships between the sequence of alpha-synuclein and its membrane affinity, fibrillization propensity, and yeast toxicity. J. Mol. Biol. 366, 1510–1522 10.1016/j.jmb.2006.12.044 17222866 PMC1868670

[B75] Voskoboinik I. Smyth M. J. Trapani J. A. (2006). Perforin-mediated target-cell death and immune homeostasis. Nat. Rev. Immunol. 6, 940–952 10.1038/nri1983 17124515

[B76] Walsh D. M. Klyubin I. Fadeeva J. V. Cullen W. K. Anwyl R. Wolfe M. S. Rowan M. J. Selkoe D. J. (2002). Naturally secreted oligomers of amyloid beta protein potently inhibit hippocampal long-term potentiation *in vivo*. Nature 416, 535–539 10.1038/416535a 11932745

[B77] Watt B. van Niel G. Fowler D. M. Hurbain I. Luk K. C. Stayrook S. E. Lemmon M. A. Raposo G. Shorter J. Kelly J. W. Marks M. S. (2009). N-terminal domains elicit formation of functional Pmel17 amyloid fibrils. J. Biol. Chem. 284, 35543–35555 10.1074/jbc.M109.047449 19840945 PMC2790984

[B78] Wickelgren I. (2004). Neuroscience. Long-term memory: a positive role for a prion? Science 303, 28–29 10.1126/science.303.5654.28a 14704404

[B79] Williams T. L. Day I. J. Serpell L. C. (2010). The effect of Alzheimer's Abeta aggregation state on the permeation of biomimetic lipid vesicles. Langmuir 26, 17260–17268 10.1021/la101581g 20923185

[B80] Williams T. L. Serpell L. C. (2011). Membrane and surface interactions of Alzheimer's Abeta peptide–insights into the mechanism of cytotoxicity. FEBS J. 278, 3905–3917 10.1111/j.1742-4658.2011.08228.x 21722314

[B81] Zampagni M. Evangelisti E. Cascella R. Liguri G. Becatti M. Pensalfini A. Uberti D. Cenini G. Memo M. Bagnoli S. Nacmias B. Sorbi S. Cecchi C. (2010). Lipid rafts are primary mediators of amyloid oxidative attack on plasma membrane. J. Mol. Med. (Berl.) 88, 597–608 10.1007/s00109-010-0603-8 20217034

[B82] Zerovnik E. Pompe-Novak M. Skarabot M. Ravnikar M. Musevic I. Turk V. (2002a). Human stefin B readily forms amyloid fibrils *in vitro*. Biochim. Biophys. Acta 1594, 1–5 10.1016/S0167-4838(01)00295-3 11825603

[B84] Zerovnik E. Turk V. Waltho J. P. (2002b). Amyloid fibril formation by human stefin B: influence of the initial pH-induced intermediate state. Biochem. Soc. Trans. 30, 543–547 12196133 10.1042/bst0300543

[B85] Zerovnik E. Zavasnik-Bergant V. Kopitar-Jerala N. Pompe-Novak M. Skarabot M. Goldie K. Ravnikar M. Musevic I. Turk V. (2002c). Amyloid fibril formation by human stefin B *in vitro*: immunogold labelling and comparison to stefin A. Biol. Chem. 383, 859–863 10.1515/BC.2002.092 12108553

[B83] Zerovnik E. Skarabot M. Skerget K. Giannini S. Stoka V. Jenko-Kokalj S. Staniforth R. A. (2007). Amyloid fibril formation by human stefin B: influence of pH and TFE on fibril growth and morphology. Amyloid 14, 237–247 10.1080/13506120701461137 17701471

